# Sensory weighting of position and force feedback during pinching

**DOI:** 10.1007/s00221-023-06654-1

**Published:** 2023-06-29

**Authors:** Jinne E. Geelen, Frans C. T. van der Helm, Alfred C. Schouten, Winfred Mugge

**Affiliations:** BioMechanical Engineering, Delft University of Technology, Mekelweg 2, Delft, 2628 CD The Netherlands

**Keywords:** Sensory weighting, Sensory integration, Pinching, Motor control, Proprioception, Fingers

## Abstract

Human hands are complex biomechanical systems that allow for dexterous tasks with many degrees of freedom. Coordination of the fingers is essential for many activities of daily living and involves integrating sensory signals. During this sensory integration, the central nervous system deals with the uncertainty of sensory signals. When handling compliant objects, force and position are related. Interactions with stiff objects result in reduced position changes and increased force changes compared to compliant objects. Literature has shown sensory integration of force and position at the shoulder. Nevertheless, differences in sensory requirements between proximal and distal joints may lead to different proprioceptive representations, hence findings at proximal joints cannot be directly transferred to distal joints, such as the digits. Here, we investigate the sensory integration of force and position during pinching. A haptic manipulator rendered a virtual spring with adjustable stiffness between the index finger and the thumb. Participants had to blindly reproduce a force against the spring. In both visual reference trials and blind reproduction trials, the relation between pinch force and spring compression was constant. However, by covertly changing the spring characteristics in catch trials into an adjusted force-position relation, the participants’ weighting of force and position could be revealed. In agreement with previous studies on the shoulder, participants relied more on force sense in trials with higher stiffness. This study demonstrated stiffness-dependent sensory integration of force and position feedback during pinching.

## Introduction

Accurate control of hands and fingers is essential for many activities of daily living. Everyday tasks such as tying a knot, writing a note, and playing the piano require dexterity and involve many degrees of freedom of the fingers. Adaptation of internal models is essential for human motor control, i.e. control signals are adjusted due to dynamics and loads (Shadmehr and Mussa-Ivaldi 1994; Ostry and Feldman 2003). Our brain continuously integrates sensory signals to obtain information on the changing loads. When controlling finger movements, the CNS has to cope with many challenges such as redundancy of muscles by balancing the recruitment of intrinsic and extrinsic muscles (Dupan et al. 2018). The CNS establishes the right balance based on the limb state and its interactions with the environment (Gardner and Costanzo 1981; Shadmehr and Mussa-Ivaldi 1994). Interaction with our environment requires coordination of movements based on accurate interpretation of sensory feedback originating from multiple modalities, including vision, audition, proprioception and touch (van Beers et al. 1996, 1999).

Previous studies targeting proprioception for upper limb control primarily focus on proximal joints (Okorokova et al. 2020). In the shoulder, sensory weighting has been found between position and force feedback when handling compliant loads. This weighting was shown to be independent of task instruction (Mugge et al. 2009). However, van Beek et al. (2016) did not find integration of force and position information in a multi-joint task in which participants navigated their hand to the center of a weak force field. Proximal and distal joints differ fundamentally in their respective functions, biomechanical properties and somatosensory organs, especially in the upper limbs. Schellekens et al. (2021) found separate shoulder-elbow and wrist-fingers clusters in the human primary motor cortex and primary somatosensory cortex based on non-rigid population response fields and Louvain modularity for a motor control task. Likewise, Goodman et al. observed a different proprioceptive representation of arm movements compared to hand movements in cortical recordings on non-human primates. Thus, differences in sensory requirements between proximal and distal joints may lead to different proprioceptive representations of fine motor skills in the sensorimotor cortex (Goodman et al. 2019). Therefore, findings at proximal joints, such as the shoulder, cannot be directly transferred to more distal joints, such as the digits.

Grasping is a well-studied task involving the digits that contains the movement of the thumb and finger towards the surface of an object (Smeets and Brenner 1999). Typically, studies on grasping investigate behaviours up until initial contact and do not extend to the subsequent phase of interaction, after picking up the object (Jeannerod 1986; Smeets et al. 2019; Flint et al. 2020). Contact between the finger and the object is required to study the interaction between internal and external forces. Both proprioceptive and tactile cues contribute to the perception of softness in pinching (Friedman et al. 2008). The neuromuscular system is a dynamic system in which forces have a position dependency (Ostry and Feldman 2003; Massimiliano 2011). The current study investigates the sensory integration of proprioceptive inputs to the CNS while maintaining a contact force between the object and the fingers, i.e. pinching.

Reaching studies have proposed that the integration of multiple sensory modalities, mostly between vision and proprioception, follows Bayesian theory (Yuille and Bülthoff 1996; van Beers et al. 1999; Körding and Wolpert 2004). Inputs from different sensory sources are weighted according to their relative reliability (van Beers et al. 1999; Körding and Wolpert 2006; Chambers et al. 2018; Massimiliano 2011). For object manipulation proprioception and touch are vital senses in movement planning (Delhaye et al. 2018). These senses cover internal and external forces and can be divided into two modalities: position and force feedback. Tasks depending on position feedback require accurate positioning of the fingers such as buttoning a shirt or typing. Whereas force feedback ensures the holding, gripping or squeezing of an object, such as eating grapes or using a scissor. Both modalities originate from the tactile mechanoreceptors in the skin (touch and pressure) and proprioceptors in the muscles (muscle force and muscle lengthening) (Mugge et al. 2012).

We hypothesize that the weighting of sensory inputs from the fingers varies based on changing requirements across diverse situations, such as pinching objects of different stiffness. We expect the weighting of force feedback to increase with object stiffness during pinching similar to the stiffness-dependent sensory weighting of force and position previously found in the shoulder (Mugge et al. 2009). When interacting with a stiffer object, a change in position (e.g., smaller aperture between thumb and index finger when pinching) will result in a larger change in force. Hence, we expect force feedback to be weighted more on stiff objects (higher forces), and position feedback to be weighted more on soft objects (smaller forces). The optimal combination of both force feedback and position feedback depends on their certainties. (Un)certainty about the sensory signals from either modality can influence the relative weighting between them. Thus, we expect to find that proprioceptive weighting exists for the fingers and that the ratio between the modalities varies with stiffness.

## Methods

### Participants

Eleven healthy volunteers participated in the study. Informed consent was obtained from all participants prior to participation. The experiment was approved by the Human Research Ethics Committee of the Delft University of Technology (#912). Eventually, one participant was excluded from the study due to technical issues with the equipment, leaving ten participants for analyses (mean age $27.2 \pm 4.6$ years, five men, five women, one left-handed).

### Experimental setup

**Fig. 1:**
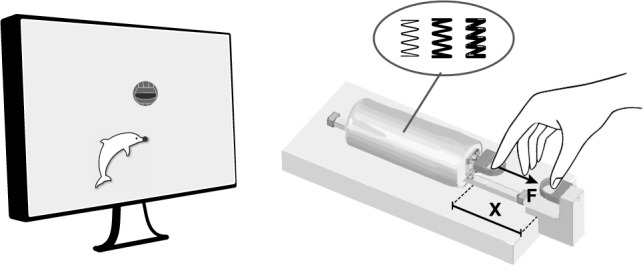
Schematic overview of the experimental setup. The finger hold for the thumb was fixed to the base, and the finger hold for the index finger was connected to the haptic manipulator. The manipulator mimicked a virtual spring with adjustable stiffness. Increasing the pinch force $(F)$ decreased the distance between the finger holds $(X_{TI})$. The target force (height of the ball) and the applied force (height of the dolphin) were presented on a screen in front of the participant. The participant was instructed to align the circle on the dolphin’s rostrum with the highlighted stripe on the ball by pinching the fingers

Participants were comfortably seated on a chair in front of a haptic manipulator, see Fig. 1. The index finger and thumb of the dominant arm were placed in the finger holds of the manipulator. The manipulator measured the pinch force (Loadcell LSB205, FUTEK) and the position of the index finger hold (LVDT, Schaevitz 2000 LCIT), with a measurement accuracy of 0.05 N and 0.03 mm, respectively (Fritz et al. 2004). The manipulator was actuated with a Maxon RE35 motor of 90W (Maxon, Sachseln, Switzerland). The applied force was controlled based on the position of the finger hold via a custom-made model running on a PLC (M1 controller module by Bachmann electronic GmbH).

To participants, the manipulator felt like they were pinching a spring. The index finger, holding the movable finger hold, with the tip pointing down opposed the thumb to create a precision pinch. A cover over the manipulator prevented direct vision of the hand. A 27-inch monitor, at a distance of approximately 1 m, provided visual feedback on the target force and the applied pinch force. The aperture was not visually fed back. The scale of the feedback was constant (force per pixel). Participants held a response button in their non-dominant hand. Pressing the button triggered a recording of 100 ms of applied force and the pinch position (measured aperture between thumb and index finger).

### Experimental procedure

Each participant performed three sixty-trial blocks. In every block, the haptic manipulator rendered a specific stiffness, see Table 1. The blocks were presented in a fixed order, starting with the lowest stiffness and ending with the highest. The participants were requested to repeatedly produce the target force and press the response button when they had attained the intended force. Participants were instructed to maintain a constant force level during the button press (i.e., to perform a force task). The zero-length of the springs was set such that for every stiffness the target pinch force resulted in an aperture of 20mm between thumb and index finger, in the middle of the 40mm operating range of the manipulator. The maximum required target force was 5N to ensure that all participants (with variable muscle strengths) were able to complete the experiment without fatiguing.

**Table 1:** Stiffness levels of the three blocks, the corresponding target force, and the position at the target force

Stiffness		Spring (N/m)	Target force (N)	Position (m)
$k_1$	Low	20	0.4	0.02
$k_2$	Medium	100	2	0.02
$k_3$	High	250	5	0.02

Before every block, participants familiarized themselves with the stiffness in a training session of at least 15 trials, including visual feedback of the applied force. In each sixty-trial block, the participants executed 30 duos of alternating trials: trials with visual feedback (reference trials) were alternated with trials without visual feedback (either blind or catch trials). To reveal the weighting of the participants out of each two trials without visual feedback one was a blind trial and one was a catch trial in random order. In a catch trial, the (virtual) regular spring was replaced by an adjusted spring. The three trial types are elaborated on in Table 2.

**Table 2:** The three trial types and their visual and dynamical properties

Trial type	Visual feedback	Force-position relation
Reference	Participants get visual feedback of the applied pinch force	The force and position of the fingertips are linearly related mimicking a regular spring
Blind	Participants do *not* get visual feedback	The force and position of the fingertips are linearly related, as in the reference trials
Catch	Participants do *not* get visual feedback	The relation between force and position of the fingertips is adjusted to reveal sensory weighting

The aperture required to reach a target force is defined by the target force and the stiffness of the spring, $k_i \; (i=1...3)$. The compression of the spring, $X$, is equal to the initial aperture between thumb and index finger, $X_{0}$, minus the aperture during pinching, $X_{TI}$. The spring is regular for reference trials and blind trials, in this case, the force, $F_{\text {regular}}$, is proportional to the spring compression:

1 $$\begin{aligned} F_{{\text {regular}},i} = k_i \cdot X. \end{aligned}$$

**Fig. 2:**
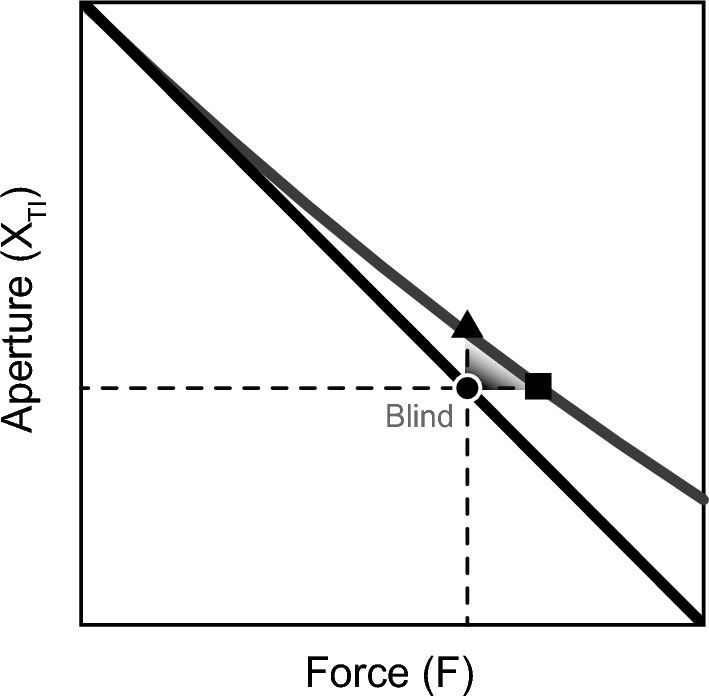
Participants were instructed to match the target force (horizontal axis) by pinching their thumb towards their index finger decreasing the aperture (vertical axis). The manipulator mimicked a regular spring (black line; reference and blind trials; Eq. 1); in catch trials, the spring was replaced with an adjusted spring (grey line; catch trials; Eq. 2). When the participant would apply the same force during blind and catch trials they will end up with a larger aperture during the catch trials (triangle). When they match the pinching aperture they will end up applying a larger force during the catch trials (square). In catch trials, they will integrate force and position sense, and end somewhere in between the triangle and square

During catch trials the spring is covertly changed to an adjusted spring, see Fig. 2. The relation of the adjusted spring force, $F_{\text {adjusted}}$, can be described by:

2 $$\begin{aligned} F_{{\text {adjusted}},i} = \left( k_i + \frac{\delta \cdot k_i^2 }{F_{{\text {target}},i}} \cdot X \right) \cdot X. \end{aligned}$$

By design, the adjusted spring requires a $10\%$ ( $\delta = 0.1$ ) higher force than the target force, $F_{{\text {target}},i}$ to achieve the same desired aperture (20mm). When a participant would keep the force constant, the resulting spring displacement will be smaller, resulting in a larger aperture. On the other hand, in the scenario where a participant matches the aperture, the resulting force will be higher. When participants internalise the spring characteristics, they will integrate the signals from both sensory modalities and end in between both scenarios. Therefore, covertly changing the spring to an adjusted spring reveals sensory weighting.

### Data analysis

#### Preprocessing

For each trial, the response button triggered the recording, and the aperture and force were determined from the recorded signals (averaged over 50 ms before till 50 ms after button press). One (reference) trial was discarded as the button was unintentionally pressed too early, i.e. within 50 ms from the start of the trial. The total dataset thus consists of 1799 trials instead of 1800: ten participants who performed sixty trials in each of the three stiffness conditions. The dataset is available via 4TU. Centre for Research Data (10.4121/21400218).

#### Force weighting factor

**Fig. 3:**
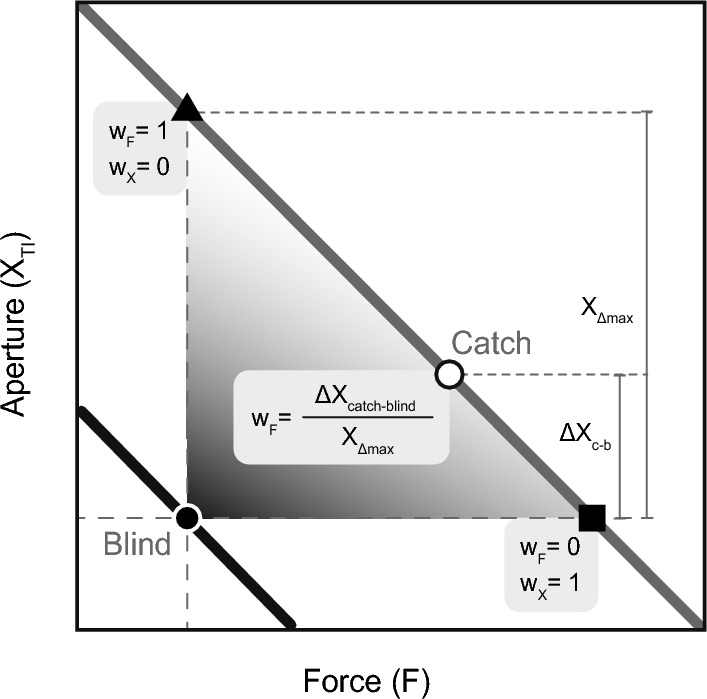
An outline of the construction of the weighting factors in a close-up view of Fig. 2. When the participant would apply the same force during blind and catch trials (triangle), they allocate all weight to the force weighting factor and none to the position weighting factor. When they match the pinching aperture (square), they allocate all weight to the position weighting factor and none to the force weighting factor. In catch trials, they will integrate force and position sense, and end somewhere in between the triangle and square along the grey line (Eq. 2). To calculate the matching force weighting factor the aperture difference between blind and catch trials is normalized to the theoretical maximal case, the difference between the blind trial and the triangle. Accounting for the inconsistent blind aperture and enabling comparison of the outcomes between participants and between stiffness conditions

For each stiffness level and for each participant, the apertures were averaged over all repetitions grouped by trial type. The difference between the apertures during the blind and catch trials ($\Delta X$) was calculated. $\Delta X$ Was normalized to the theoretical maximum value, the aperture difference for an equal force in regular and adjusted spring. This results in a relative outcome measure: the force weighting factor ($W_{\text {F}}$). Testing $W_{\text {F}}$ accounts for the variable bias between participants and stiffness conditions in reproduced force during blind trials. Figure 3 shows the relation between the absolute and relative outcome measures. The normalized aperture during catch trials revealed the weighting of force feedback ($W_{\text {F}}$), see Fig. 3.

#### Statistics

A significance level of 0.05 was maintained for the statistical tests. *p* values in post-hoc analysis were adjusted using the Benjamini and Hochberg (1995) multiple testing correction method to control for the false discovery rate (FDR). Outliers were checked via the rstatix package (version 0.7.0, by Alboukadel Kassambara). The data were assessed on normality by Shapiro–Wilk’s test. A Friedman test checked for stiffness effects on $W_{\text {F}}$ followed by a post-hoc pairwise Wilcoxon signed-rank test.

## Results

The data were normally distributed ($p > 0.2$), and no extreme outliers were found.

**Fig. 4:**
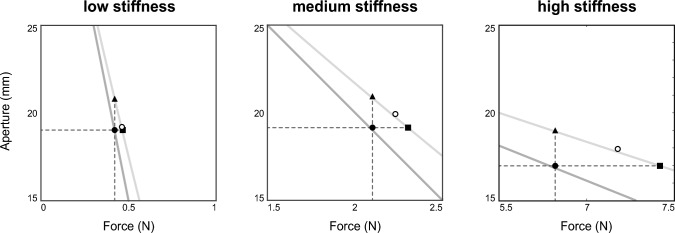
Pinching aperture and force for the three stiffness levels averaged over all participants. The solid lines indicate the theoretical force-aperture relation of the springs. The filled circle indicates the reproduced force in the blind trials; the open circle indicates the reproduced force in the catch trials. The dashed reference lines show the theoretical extreme apertures for the catch trials

**Fig. 5:**
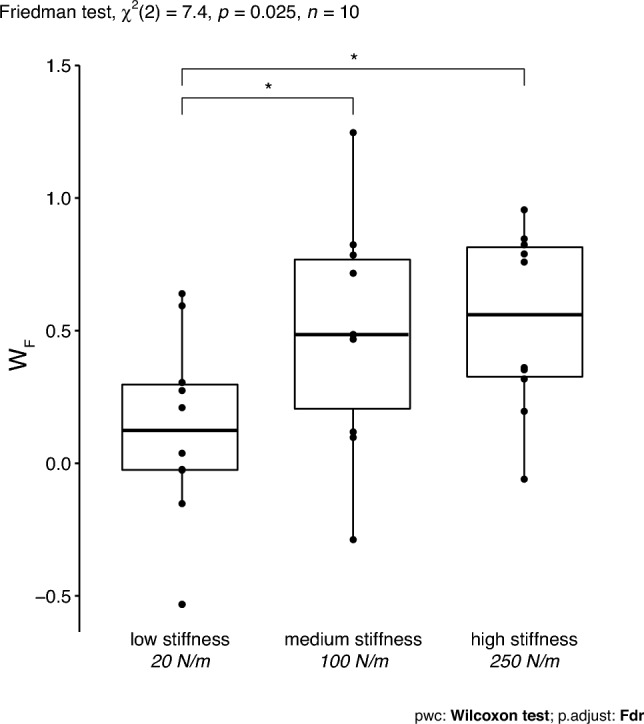
Boxplots for the weighting factor of force feedback. $W_{\text {F}}$ is significantly lower for the lowest stiffness level compared to both the medium and the high stiffness conditions

Figure 4 presents the final apertures for the blind and catch trials in the three stiffness conditions. During catch trials (open circle), participants ended up between the triangle and square, which indicates the sensory weighting, see also Fig. 2. For the low stiffness condition, the aperture in catch trials was nearest to the square, indicating that participants relied relatively more on position feedback. For the medium and high stiffness conditions, participants ended somewhere in between the triangle and square, thus relying on a combination of force and position feedback. The sensory weight for force, $W_{\text {F}}$, is equal to the ratio between the apertures normalized to the aperture difference at the target force, see Fig. 5. The force weighting factor was significantly different for different stiffness levels, $\chi ^{2}(2) = 7.4$ ($p = 0.025$), with a moderate effect size ($W = 0.37$). Pairwise Wilcoxon signed rank test between the stiffness levels revealed that $W_{\text {F}}$ was significantly lower for the low stiffness condition, ($p < 0.05$). This indicates that the weighting of the sensory modalities varied with stiffness.

## Discussion

When pinching compliant objects, both position and force sense provide task-relevant information. Our results confirm that weighting of force feedback increases with object stiffness during pinching, similar to the stiffness-dependent sensory weighting of force and position previously found in the shoulder (Mugge et al. 2009). Participants relied more on force sense when interacting with a stiffer object. This study shows that a stiffness-dependent multi-sensory integration is present for joints in fine motor skills such as pinching.

Sensory feedback can be grouped into function-based modalities, including the modalities relevant to our experiment: vision, proprioception, and touch. Touch and proprioception are umbrella modalities containing overlapping submodalities. We studied the weighting within proprioception and touch between their submodalities position and force, two basic mechanical concepts. Another way of dividing submodalities is based on the mechanoreceptive afferents that they originate from Saal and Bensmaia (2014). Kim et al. (2015) managed to separate proprioceptive manipulations and cutaneous stimulations in primates and showed multimodal processing of digit representation in the primary somatosensory cortex. Little is known about where the submodalities are integrated into the medial lemniscal pathway and how this benefits naturalistic contexts (Saal and Bensmaia 2014). A combination of multiple mechanoreceptive afferents leads to a percept of the internal and external forces at play. However, recordings in area 2 of the somatosensory cortex by Okorokova et al. (2020) suggest that cutaneous signals obscure proprioceptive signals rather than complement them.

Friedman et al. studied the perceived softness of an object when actively, passively, and indirectly pressed with the index finger. They concluded that participants use both tactile and kinesthetic sensory cues to assess soft versus hard objects. Objects were classified as soft if the stiffness was lower than the stiffness of the finger. This suggests that the classification of softness depends on whether the object conforms to the body, and thus that tactile feedback about object deformation is sufficient to estimate softness (Friedman et al. 2008). This conclusion is in line with our findings, that the weight of position feedback was the highest for the spring with the lowest stiffness.

To prevent an effect of sensorimotor memory all participants performed the conditions in ascending order of stiffness. Sensorimotor memory is the influence of trial history on object weight perception, as previously shown for subsequent trials of pinching by Johansson and Westling (1984). Van Polanen and Davare showed that the perception of light objects is influenced by the previous weight: the object feels lighter when a heavy object was previously lifted compared to when it was preceded by a light one. This perceptual bias was only found if the current lift was light, but not heavy (van Polanen and Davare 2015). In addition, long breaks (at least 15 min) between the blocks were introduced to avoid habituation and fatigue. In case of habituation, trials early in a block would be different from trials later in a block. This effect is not observed for any of the conditions in our study. Fatigue may have lowered the effect size though, as force variability is known to increase in variance during force production by the index finger (Singh et al. 2010). This variance increase would result in a decrease of force weighting in the last block, contrasting the effect we observed. Hence, we conclude that the observed change in weighting is due to an effect of stiffness.

The current experiment design was not optimized to study the structural deviation from the target force during blind trials. Yet, our data showed a force reproduction error in high stiffness trials. Participants overshot the target in high stiffness blind trials using more force than during reference trials, see Appendix B. Results from previous research at the shoulder contradict ours at the finger as they show an undershoot for increasing force targets (Onneweer et al. 2016).

### Maximum likelihood estimation

**Fig. 6:**
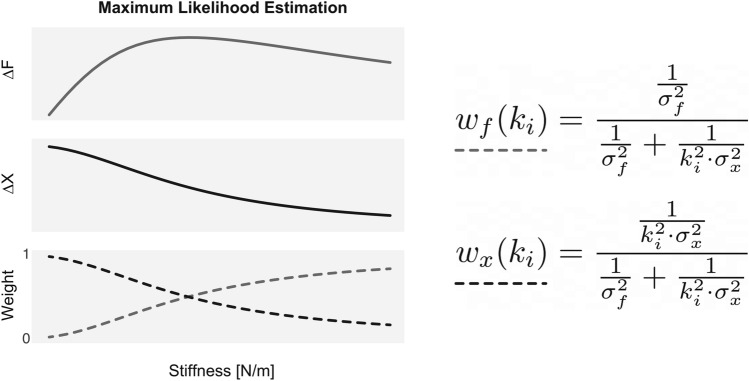
An example of an estimated MLE-based weighting factor profile for one of the participants. The weight factors are calculated with the formulas at the right using the variance of the force feedback $\sigma ^{2}_{\text {f}}$ and variance of the aperture feedback $\sigma ^{2}_{x}$. The top panel shows the predicted change in force between the regular and adjusted spring ($\Delta F$) over the increasing stiffness of the springs. The middle panel shows the predicted change in aperture/position ($\Delta X$). The bottom panel represents the crossing force weight factor $w_{\text {f}}$ and position weight factor $w_x$

Human sensory integration can be predicted by maximum likelihood estimation (MLE) (Mugge et al. 2009; Körding and Wolpert 2006). An MLE model predicts the optimal integration of the proprioceptive sensory signals based on the relative uncertainty of the sensory signals, indicated by their variance. With stiffness, force and position are related. The stiffness-dependent ($k_i$) weighting of the force feedback ($w_{\text {f}}$) is inversely proportional to the variance of sensory force feedback ($\sigma _{\text {f}}^2$). Similarly, the weighting of position feedback ($w_x$) is inversely proportional to the variance of sensory position feedback ($\sigma _x^2$) multiplied by the squared stiffness to transform both modalities to force units. The weights are normalized such that $w_{\text {f}} + w_x = 1$. Every participant has their own crossover point where the force and position weights are equal. An example of an MLE-based weighting factor profile was made for one of the participants, see Appendix A (Fig. 6). One can estimate the theoretical crossover point for a participant by evaluating their specific variance profile for the two modalities in extreme situations (no stiffness and infinite stiffness) (Mugge et al. 2009; Körding and Wolpert 2006).

When motor behaviour is involved, the role of sensory variance is complex. While relative signal variance influences visuo-proprioceptive weighting (van Beers et al. 1999; Block and Sexton 2020), weighting may also be affected by the computation being performed, i.e. movement vector vs. motor command (Sober and Sabes 2003, 2005); by the dimension of space being estimated (van Beers et al. 2002); and by attention and cognitive factors (Block and Bastian 2010; Warren and Schmitt 1978; Welch and Warren 1980). Although the reaching literature has found support for MLE integration of vision and proprioception, there are examples of weighting being affected by parameters other than signal variance. Intermodal (Visual and proprioceptive) sensory information can be reweighted by conscious effort (Warren and Schmitt 1978; Block and Bastian 2010), while intramodal (proprioceptive) sensory weighting did not significantly change with task instructions (Mugge et al. 2009). Various stages of motor planning require distinctive strategies of sensory integration (Sober and Sabes 2003, 2005). The current study allowed subjects to take time to correct their aperture after the reach achieving a semi-static posture to maintain constant computations and dimensions across stiffness conditions.

van Beers et al. (2011) show that reweighting also occurs per trial, based on provided feedback. Nevertheless, blind and catch trials lack visual feedback in our experiment design. Participants were instructed to keep their eyes focused on the screen to ensure they were not reweighting during the reference trials. Another possibility of feedback during our experiments could have been the limited range of the manipulator. In the highest stiffness condition, some participants applied such high forces that they were close to bringing the finger holds together. None of the participants reported that their fingers touched. However, we did not check for contact. Visual feedback during our experiments was negligible, and although reweighting based on tactile feedback could have occurred in some trials, an MLE-based profile could still be expected.

### Limitations

The sensory weighting of the index finger was isolated in this experiment during a pinching task. This task could translate to activities involving a precision grip such as sprinkling a pinch of salt, changing a light bulb, or picking up small objects. This contrasts with grasping studies that usually include all the fingers and thus translate to other activities of daily life.

Visual feedback improved precision as blind and catch trials showed higher variances of aperture for all stiffness blocks compared to reference trials. Within reference trials, the aperture spread was largest in the low stiffness block. This is likely due to the equal scaling of the visual feedback across stiffness blocks. For a particular visual error (force), the aperture error is larger for low stiffness than for high stiffness, explaining the higher aperture variance in the low stiffness block. Whether the scaling of visual feedback affected weighting is unknown.

In our experiments, the three stiffness blocks were equal for all participants. However, their strength varied considerably and where some indicated the high stiffness level was challenging, others could probably have handled higher stiffness without fatiguing. The stiffness range of the manipulator was limited, thus reducing the experimental design options. A setup with a more extensive range would allow for additional stiffness blocks to fit a wider range of individual crossover points.

Despite the normal distribution of the data, non-parametric tests were used to account for the small sample size. Friedman test is often seen as the non-parametric counterpart of repeated-measures ANOVA. However, it is a generalization of the sign test that handles both normal as well as many non-normal distributions and offers lower statistical power than repeated-measures ANOVA (Zimmerman and Zumbo 1993).

## Conclusion

We revealed sensory weighting in the control of fingers during fine motor skills. Similar to the previously found results in the shoulder, the ratio between the modalities varied with stiffness. We found a significantly different weighting for low stiffness (20N/m) compared to medium and high stiffness conditions (100N/m and 250N/m, respectively). This study demonstrated stiffness-dependent sensory integration of force and position feedback during pinching.

## Figures and Tables

**Fig. 7:**
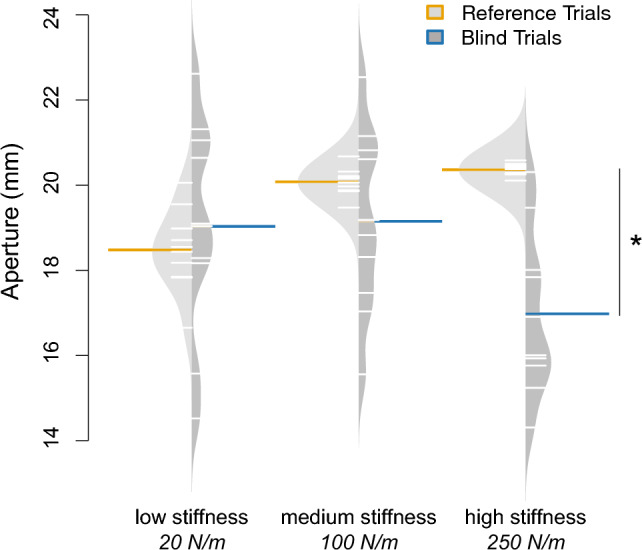
The distribution of the mean apertures of all participants during reference trials (light grey, mean in orange) next to blind trials (dark grey, mean in blue)

## Data Availability

The dataset is available via 4TU. Centre for Research Data (10.4121/21400218). This article is licensed under a Creative Commons Attribution 4.0 International License, which permits use, sharing, adaptation, distribution and reproduction in any medium or format, as long as you give appropriate credit to the original author(s) and the source, provide a link to the Creative Commons licence, and indicate if changes were made. The images or other third-party material in this article are included in the article’s Creative Commons licence unless indicated otherwise in a credit line to the material. If material is not included in the article’s Creative Commons licence and your intended use is not permitted by statutory regulation or exceeds the permitted use, you will need to obtain permission directly from the copyright holder. To view a copy of this licence, visit http://creativecommons.org/licenses/by/4.0/.

## Appendix A: Maximum likelihood estimation

One of the participants performed an extra experiment to estimate a personal MLE profile. Two additional conditions were performed to resemble the extreme cases of $w_{\text {f}} = 1$ with $w_x = 0$ and the other way around to estimate their uncertainties. The $w_x = 0$ case included a fixed aperture by locking the finger handle to 20 mm. This aperture represents the target in the main experiment to ensure a similar posture. The participant was provided with a force task and force feedback, equal to the main experiment in this study. The $w_{\text {f}} = 0$ situation was technically not feasible with our manipulator, it could not create a perfect force-less environment. Thus the lowest stiffness possible was chosen (0.1 N/m$^2$) to approach $w_{\text {f}} = 0$. The task for the participant changed to a position task with position feedback. The $\sigma ^{2}_{\text {force}}$ in this example was found to be 0.4 N$^2$ and the $\sigma ^{2}_{\text {aperture}}$ = 13.9 mm$^2$. The cross-over for this participant probably approaches 288 N/m, considering the limited possibilities for accurate variance estimation this guess should be interpreted carefully. If the actual intersection of $w_{\text {f}}$ and $w_x$ are near this course estimation it would imply that the chosen stiffness levels did not reach the intersection point for this participant. Future experiments require a more advanced manipulator that allows for a zero-stiffness environment, higher stiffness levels, and a wider range of apertures.

In Fig. 6, the predicted change in force between the regular and adjusted spring ($\Delta F$) and the predicted change in aperture ($\Delta X$) paths are switched compared to the predictions of the MLE by Mugge et al. (2009). This is due to the difference in experimental design. In this study, the aperture is constant over all stiffness levels. In their study, the force was constant over all stiffness levels.

## Appendix B: Force reproduction error

In the absence of visual feedback, during blind and catch trials, participants could overestimate or underestimate their applied pinch force, a known phenomenon called force reproduction error (Onneweer et al. 2016). When comparing the mean aperture of all participants for reference trials and blind trials a systematic error appears, see Fig. 7 in style of (Kampstra 2008). A two-way repeated measures ANOVA was performed and revealed an effect between trial type and stiffness on the aperture between reference and blind trials *F*(2, 18) = 13.356, $p < 0.001$. For the high stiffness conditions, participants overshot the target during blind trials using more force than during reference trials. This indicates that they underestimated the applied forces during blind trials. Pairwise comparisons showed that the aperture significantly differed between reference and blind trials, indicating a bias in the high stiffness condition ($p < 0.001$).
